# Ohio Primary Health Care Providers’ Practices and Attitudes Regarding Screening Women With Prior Gestational Diabetes for Type 2 Diabetes Mellitus — 2010

**DOI:** 10.5888/pcd11.140308

**Published:** 2014-12-04

**Authors:** Loren Rodgers, Elizabeth J. Conrey, Andrew Wapner, Jean Y. Ko, Patricia M. Dietz, Reena Oza-Frank

**Affiliations:** Author Affiliations: Elizabeth J. Conrey, Centers for Disease Control and Prevention, Atlanta, Georgia, and Ohio Department of Health, Columbus, Ohio; Andrew Wapner, Ohio Department of Health, Columbus, Ohio; Jean Y. Ko, Patricia M. Dietz, Centers for Disease Control and Prevention, Atlanta, Georgia; Reena Oza-Frank, Research Institute at Nationwide Children’s Hospital, Columbus, Ohio, and Ohio State University, Columbus, Ohio.

## Abstract

**Introduction:**

Gestational diabetes mellitus (GDM) is associated with a 7-fold increased lifetime risk for developing type 2 diabetes mellitus. Early diagnosis of type 2 diabetes is crucial for preventing complications. Despite recommendations for type 2 diabetes screening every 1 to 3 years for women with previous diagnoses of GDM and all women aged 45 years or older, screening prevalence is unknown. We sought to assess Ohio primary health care providers’ practices and attitudes regarding assessing GDM history and risk for progression to type 2 diabetes.

**Methods:**

During 2010, we mailed surveys to 1,400 randomly selected Ohio family physicians and internal medicine physicians; we conducted analyses during 2011–2013. Overall responses were weighted to adjust for stratified sampling. Chi-square tests compared categorical variables.

**Results:**

Overall response rate was 34% (380 eligible responses). Among all respondents, 57% reported that all new female patients in their practices are routinely asked about GDM history; 62% reported screening women aged 45 years or younger with prior GDM every 1 to 3 years for glucose intolerance; and 42% reported that screening for type 2 diabetes among women with prior GDM is a high or very high priority in their practice.

**Conclusion:**

Because knowing a patient’s GDM history is the critical first step in the prevention of progression to type 2 diabetes for women who had GDM, suboptimal screening for both GDM history and subsequent glucose abnormalities demonstrates missed opportunities for identifying and counseling women with increased risk for type 2 diabetes.

## Introduction

Gestational diabetes mellitus (GDM) is glucose intolerance that initiates or is first diagnosed during pregnancy, excluding overt diabetes, and affects from 2% to 10% of all pregnant women annually in the United States (1,2). Approximately one third of women with GDM will be identified as having diabetes or glucose intolerance at their 6-week postpartum visit (3–5). Women with diagnosed GDM are at as much as an 84% increased risk for GDM during subsequent pregnancies (6), and the risk for developing type 2 diabetes mellitus during their lifetime is approximately 7-fold greater than that for women with normoglycemic pregnancies (7). Prior analyses suggest that cumulative incidence of type 2 diabetes ranges from 20% to 60% within 10 years of GDM diagnosis, plateauing thereafter (8,9). Without appropriate screening, type 2 diabetes often remains undiagnosed as a result of insidious and asymptomatic clinical progression until onset of secondary complications, including hypertension, heart disease, stroke, retinopathy, and renal failure. Furthermore, uncontrolled type 2 diabetes complicates subsequent pregnancies and is associated with birth defects and birth-related illnesses in the child (10).

The American Diabetes Association (ADA) recommends that women with prior GDM be screened for type 2 diabetes at 6 to 12 weeks postpartum and at least every 3 years thereafter by testing fasting plasma glucose, oral glucose tolerance, or hemoglobin A1c (2). The American College of Obstetricians and Gynecologists (ACOG) recommends postpartum screening for women who had GDM, and refers to ADA recommendation for subsequent screening (11).

In the United States, prevalence of postpartum screening is reported to be suboptimal for women with prior GDM (12–14). Women receive preventive medical services from various practitioners, and most prior work documenting screening has focused on care provided by obstetrician/gynecologists (OB/GYNs) and midwives. In Ohio, 70% of OB/GYNs and 50% of midwives reported almost always screening for type 2 diabetes mellitus during the postpartum visit (15,16). In 2011, Blatt et al analyzed data from a laboratory database and found that only 19% of 23,299 women with GDM-affected pregnancies were tested for diabetes within 6 months after delivery (17). Follow-up screening may also be suboptimal; one study reported that during the 5 years after the postpartum period, 41% of women with prior GDM had not been tested for diabetes by an OB/GYN, family practitioner, or internal medicine physician (18). Few studies have focused exclusively on general health practitioners; therefore, we surveyed Ohio primary health care providers to understand their practices and attitudes related to ascertaining GDM history and screening for subsequent type 2 diabetes as part of a public health effort to improve type 2 diabetes screening rates for women with a GDM history.

## Methods

We developed a questionnaire with 25 questions related to clinical specialization; patient demographics; and knowledge, attitudes, and practices regarding GDM, screening for GDM history, and subsequent screening for type 2 diabetes; other findings from this survey were published previously (15,16,19). After pilot-testing the questionnaire for clarity with 4 family physicians, we used data from the Ohio eLicense Center to randomly sample 700 of 2,253 physicians licensed in Ohio with “family medicine” or “family practice” self-reported as specialties and 700 of 4,726 with “internal medicine” or “general practice” reported (20). We mailed paper questionnaires up to 2 times during 2010, preceded by a postcard announcement and interposed by a postcard reminder, with an option to respond online. Nonrespondents were sent a final request by e-mail. We subsequently deemed respondents ineligible if they were not treating female patients, worked primarily in a nursing home or long-term care facility, did not routinely deliver primary care, or if they were hospitalists, retired, or primarily practicing outside Ohio, as indicated by notes on returned survey reminders, direct communication from respondents, or survey responses. Respondents were also deemed ineligible if survey responses indicated that questions regarding screening for glucose intolerance do not apply to their practice.

### Data analysis

Response options to attitude questions were “strongly agree,” “somewhat agree,” “somewhat disagree,” “strongly disagree,” and “unsure”; responses of “somewhat disagree” were grouped with “strongly disagree.” Response options to the question about priority level for screening for type 2 diabetes were “very high,” “high,” “moderate,” “low,” and “very low”; responses of high were grouped with “very high,” and responses of “low” were grouped with “very low.” All choices were mutually exclusive. We used Microsoft Excel 2010 (Microsoft Corp) and SAS 9.2, (SAS Institute, Inc) for data analysis, including Pearson’s chi-square tests to compare contingency tables of categorical variables, 2-proportion z-tests for proportions, and 2-sample Student’s *t* tests for continuous variables. We set significance at α = .05. Because this survey design relied on independently sampled specialties, overall responses were weighted to adjust for sampling rates among specialties, and first-order Rao-Scott chi-square tests were used for comparisons of weighted responses. Missing data were excluded from response percentages; variance estimations for overall weighted percentages account for missing responses. Data were analyzed during 2011–2013.

Among 700 selected primary care physicians, 115 were deemed ineligible because they indicated that they do not treat female patients, do not routinely deliver primary care, are retired, primarily practice outside Ohio, work primarily in a nursing home or long-term care facility, or reported that questions about screening for glucose intolerance do not apply to their practice. Among the remaining 585 surveyed who were designated as eligible, 230 (39%) returned completed surveys. Among 700 surveyed internal medicine or general practice physicians, 170 were excluded because of ineligibility; 530 were eligible, and 150 (28%) of those returned completed surveys. Overall response rate was 34% (380 eligible respondents) (Figure). Of those 380 respondents, 66% were men, 74% were primarily in a private practice, and 95% reported that they do not provide prenatal care (Table 1).

**Figure:**
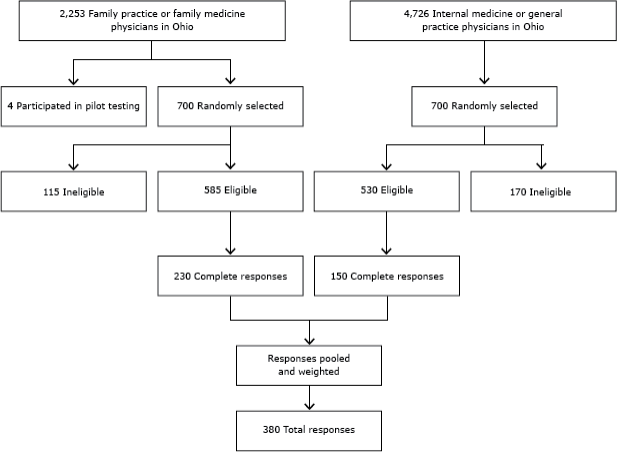
Stratified random sampling of primary health care providers surveyed regarding attitudes and practices about screening women with prior gestational diabetes for type 2 diabetes mellitus — Ohio, 2010.

**Table 1:** Characteristics and Diabetes Screening Practices of Surveyed Primary Health Care Providers in Ohio — 2010

Variable	Overall %^a^ (95% CI), N = 380
**Sex**	
Male, n (%)	250 (66)
Female, n (%)	130 (34)
**Practice location**	
Federally qualified health center	5 (3–7)
Hospital	12 (9–16)
University	5 (3–8)
Private, ≤2 physicians	34 (29–39)
Private, >2 physicians	40 (35–46)
Other	3 (1–5)
**Predominant patient population**	
Urban	23 (19–28)
Suburban	54 (48–59)
Rural	23 (19–27)
**Percentage of patients with care paid by Medicaid**	
≤25%	56 (51–61)
26%–50%	19 (15–24)
51%–75%	5 (3–8)
>75%	2 (0–4)
Accept Medicaid; do not know percentage of patients on Medicaid	2 (1–4)
Do not accept Medicaid	15 (11–19)
**Do you provide prenatal care?**	
Yes	5 (3–7)
No	95 (93–97)
**In my practice, all new female patients are asked if they have a history of gestational diabetes mellitus**	
Yes	57 (52–63)
No	43 (37–48)
**In your practice, how often are nonpregnant women aged ≤45 years, with histories of gestational diabetes mellitus screened for glucose intolerance?**	
Every 1–3 years	62 (56–67)
Dependent on risk factors	31 (26–36)
Do not provide screening	8 (5–11)

Abbreviation: CI, confidence interval.

a Stratified random sample survey design; overall percentages weighted to adjust for sampling rates among provider specialties.

The Centers for Disease Control and Prevention determined that the survey and analytic activities constituted public health practice and was exempted from IRB review.

## Results

### Screening practices and attitudes of respondents

Of all respondents, 57% reported that all new female patients in their practice are asked if they have a history of GDM; similar proportions of family physicians and internal medicine physicians reported routinely asking about GDM history (57% vs 58%, respectively; *P* = .85). Sixty-two percent of all respondents reported screening every 1 to 3 years for glucose intolerance among nonpregnant women aged 45 years or younger with a history of GDM (Table 1). Sixty-four percent of respondents strongly agreed that “GDM has long-term implications for a woman’s health”; 65% strongly agreed that “It is part of my job to help women with a history of GDM to improve their diet and exercise regularly”; 70% strongly agreed that “It is important for me to increase patient knowledge of future risk for type 2 diabetes among patients with a history of GDM”; and 71% strongly agreed that “There is a need for periodic screening for type 2 diabetes among women with a history of GDM” (Table 2). Among respondents, 42% ranked screening for type 2 diabetes among nonpregnant women with prior GDM as a high or very high priority (Table 2). Responses to these questions did not vary significantly by respondent’s sex, the demographic population served by the respondent (urban, suburban, or rural), or the percentage of patients with care paid by Medicaid (*P* > .05 for each, data not shown).

**Table 2:** Ohio Primary Health Care Providers’ Attitudes by Screening Practices for Gestational Diabetes Mellitus History — 2010

Survey Question	Overall %^a^ (95% CI), N = 380	Respondents Who Report Asking All New Female Patients About Prior GDM, % (95% CI), n = 197	Respondents Who Report Not Asking All New Female Patients About Prior GDM, % (95% CI), n = 149	*P* Value^b^
**GDM has long-term implications for a woman’s health.**				
Strongly agree	64 (59–69)	69 (62–75)	56 (48–65)	.12
Somewhat agree	28 (23–33)	24 (18–31)	34 (26–42)	
Disagree^c^	4 (2–6)	5 (1–8)	4 (1–7)	
Unsure	4 (2–6)	3 (0–5)	6 (1–10)	
**It is part of my job to help women with a history of GDM to improve their diet and increase exercise.**				
Strongly agree	65 (60–70)	75 (68–81)	53 (44–61)	.002
Somewhat agree	7 (22–32)	19 (13–25)	37 (29–45)	
Disagree^c^	6 (3–8)	5 (2–8)	8 (3–13)	
Unsure	2 (0–3)	1 (0–3)	3 (0–5)	
**It is important to me to increase patient knowledge of future risk of type 2 diabetes among patients with a history of GDM.**				
Strongly agree	70 (65–75)	77 (71–83)	60 (52–68)	.001
Somewhat agree	25 (20–29)	19 (13–25)	32 (24–40)	
Disagree^c^	3 (1–5)	4 (1–7)	4 (0–7)	
Unsure	2 (1–3)	0 (0–1)	5 (1–8)	
**There is a need for periodic screening for type 2 diabetes among women with a history of GDM.**				
Strongly agree	71 (66–75)	77 (71–83)	61 (53–69)	.001
Somewhat agree	22 (18–26)	17 (12–22)	30 (22–37)	
Disagree^c^	4 (2–6)	4 (1–7)	3 (0–6)	
Unsure	3 (2–5)	1 (0–3)	7 (2–11)	
**To what extent is screening for type 2 diabetes among nonpregnant women with a history of GDM a priority in your practice?**				
High or very high	42 (37–48)	59 (52–67)	24 (17–31)	<.001
Moderate	37 (32–42)	35 (28–42)	38 (30–46)	
Low or very low	20 (16–25)	6 (3–9)	38 (30–47)	

Abbreviations: CI, confidence interval; GDM, gestational diabetes mellitus.

a Stratified random sample survey design; overall percentages weighted to adjust for sampling rates among provider specialties.

b First-order Rao-Scott χ^2^ test.

c Includes responses of “somewhat disagree” and “strongly disagree.”

Clinician attitudes are associated with screening women with prior GDM for type 2 diabetes (Table 2). Respondents whose practices screened patients for GDM history strongly agreed more frequently than did respondents who do not routinely ascertain prior GDM diagnoses with the following: that a part of their job is to help women with prior GDM improve their diet and exercise (75% vs 53%; *P* < .001); that increasing patient knowledge of future risk for type 2 diabetes among patients with GDM history is important (77% vs 60%, respectively, *P* = .002); and that a need exists for periodic screening for type 2 diabetes among women with a history of GDM (77% vs 61%, respectively; *P* = .002). Among the respondents who ask all new female patients about GDM history, 59% ranked screening nonpregnant women with a history of GDM for type 2 diabetes as a high or very high priority, compared with 24% of respondents who do not routinely ascertain prior GDM diagnoses (*P* < .001).

Although 85% of all respondents reported counseling women with histories of GDM about physical activity, fewer (17%) referred these women to resources for increasing physical activity; counseling and referrals for nutrition were similar (Table 3). Comparing physicians whose practices screen all new female patients for GDM history with those who do not, 23% versus 10% (*P* = .003) refer them to community resources to increase physical activity, and 38% versus 14% (*P* < .001) refer these women to nutrition counseling resources, respectively.

**Table 3:** Prevalence of Gestational Diabetes Mellitus-Related Referrals and Counseling Among Primary Health Care Providers, by Screening for Gestational Diabetes Mellitus History — Ohio, 2010

Survey Question	Overall %^a^ (95% CI), N = 380	Respondents Who Report Asking All New Female Patients About Prior GDM, % (95% CI), n = 197	Respondents Who Report Not Asking All New Female Patients About Prior GDM, % (95% CI), n = 149	*P *Value^b^
**When I provide care to women with histories of GDM, I ____ (Select all that apply).**				
Counsel them about nutrition/diet	79 (74–83)	85 (80–90)	77 (70–84)	.09
Counsel them to exercise regularly/increase physical activity	85 (81–89)	87 (82–92)	86 (80–92)	.82
Refer them to a diet support group or other nutrition counseling resources in the community	27 (22–32)	38 (31–45)	14 (9–20)	<.001
Refer them to community resources to increase activity	17 (13–21)	23 (17–29)	10 (4–15)	.003

Abbreviations: CI, confidence interval; GDM, gestational diabetes mellitus.

a Stratified random sample survey design; overall percentages weighted to adjust for sampling rates among provider specialties.

b First-order Rao-Scott χ^2^ test.

## Discussion

The identification of women with prior GDM, and subsequent lifelong screening for glucose intolerance, is a critical step in preventing type 2 diabetes or identifying the disease early. Screening is particularly important because many women with prior GDM lack additional risk factors (such as obesity) and would otherwise not be screened on the basis of age alone. This study sought to characterize the practices and attitudes of primary care physicians regarding long-term screening for type 2 diabetes among women with prior GDM to aid in the development of public health interventions to increase screening rates by primary care physicians.

Rates for screening were suboptimal, with approximately half (57%) of respondents indicating that all new female patients in their practices are screened for a history of GDM, and fewer than two-thirds (62%) reporting that, every 3 years, they screen all women aged 45 years or younger with prior GDM for glucose intolerance. Similarly, Stuebe et al found that 44% of surveyed primary care providers reported asking women of reproductive age about GDM history during at least half of office visits (21). These low rates reflect substantial missed opportunities, not just for early identification of type 2 diabetes, but also for ensuring healthy future pregnancies, because women with GDM during their first pregnancy have a nearly tenfold increased risk for GDM during their second pregnancy (22). Recurrent GDM increases the risk for adverse newborn outcomes beyond those observed during an initial GDM-complicated pregnancy (23). Early ascertainment of GDM history is therefore an important first step in providing preventive care for women between pregnancies (interconception care).

One limitation of this study is that practices and attitudes related to screening for GDM history and type 2 diabetes might be overestimated if health care providers most interested in the topic responded more frequently to the survey than did providers for whom GDM and type 2 diabetes are of less interest. Our response rate was only 34%; however, surveys of primary care physicians are often completed at a rate of 40% or lower (12,21). Additionally, some nonrespondents might have been ineligible for the survey, and if so, the response rate among eligible survey recipients would have been higher. Furthermore, respondents might have selected responses they perceived to be more socially desirable. Thus, the true screening rates may be lower than reported in this survey, and these data may not be generalizable beyond clinicians practicing in Ohio.

This study assessed the care provided to women with prior GDM. Lifestyle modifications aimed at weight loss and physical activity are effective for decreasing progression to type 2 diabetes (24), and both the ADA and ACOG recommend diet, exercise, and weight management counseling for women with high risk of developing type 2 diabetes (2,11). Accordingly, most respondents reported providing counseling to improve nutrition (79%) or increase physical activity (85%) when they knew a woman had prior GDM. We also found an association between screening and physician care, with those reporting screening women for prior GDM more frequently reporting a role in helping women improve nutrition, increase physical activity, and better understand their risk for type 2 diabetes and the need for lifelong screening. Although a causal relationship between these attitudes and practices cannot be inferred from this cross-sectional survey, these findings are consistent with the Theory of Planned Behavior, which describes behavioral practices as partially dependent on a person’s attitudes (25). Attitudes that could contribute to screening practices include providers’ perceptions of patient care responsibilities. Primary care physicians and internal medicine physicians indicated that they have a role in caring for women with prior GDM (19). However, in the United States, nonpregnant women younger than 50 years of age see OB/GYNs for most preventive visits (26), and some primary care providers may rely on such specialists for ascertainment of GDM history, particularly if the clinical relevance of GDM is perceived to primarily concern pregnancy.

Transition to primary care providers after pregnancy introduces potential for discontinuity of care for women with prior GDM, particularly since a decreasing proportion of family physicians in the United States provide obstetric and maternity care (27). Health information does not always transfer between practices or health care systems. One study of providers within a single health care system found that among 772 women with prior GDM, 46% had the condition documented in the network’s tool used to manage patient care, and only 22% of primary care respondents’ intake forms included assessment of GDM history (21). The same study reported that 55% of surveyed providers cited communication gaps between primary care providers and obstetrics and gynecology care providers as a barrier to providing post-GDM follow-up care, which further underscores the importance of proactive ascertainment of GDM history (21).

Collectively, these results indicate a series of missed opportunities for preventing the progression to or early identification of type 2 diabetes. Opportunities for improving women’s health and birth outcomes include periodic blood glucose screening and equipping patients to make lifestyle changes, but these opportunities begin with early identification of GDM history. Opportunities to improve screening exist at multiple levels, including individual primary care providers (asking patients about GDM), practices/clinics (including GDM-related questions on intake forms and implementing policies to ensure screening for GDM history and type 2 diabetes), health care networks (improving documentation of GDM history; health information exchanges), and insurers (improving access to lifestyle interventions and counseling). These results also highlight a need for public health agencies to identify and address barriers that hinder comprehensive follow-up for women with prior GDM. Providers identified a need for increased resources for improving care of women with prior GDM, including local nutrition specialists, local resources for physical activity, and patient education materials (19). Additional challenges that hinder weight-reduction interventions include insurers’ unwillingness to pay for obesity mitigation programs, physicians’ limited time for patient education and monitoring, physicians’ skepticism or uncertainty about the effectiveness of diet and exercise counseling, and patients’ failure to comply (28). Public health agencies can reduce progression of GDM to type 2 diabetes by supporting interventions that address these barriers, thereby empowering physicians to reduce the long-term impact of GDM. Understanding factors that contribute to low screening rates is critical for public health interventions aimed at increasing lifelong type 2 diabetes screening during primary care visits.

Public health leaders can improve patient outcomes by fostering better integration of primary care, obstetric, maternal and child health, and chronic disease systems. In Ohio, a collaborative was formed at the outset of this study to improve lifelong health outcomes related to GDM. This group is using these findings to create patient education and other resources for women and health care providers to better understand and provide care for GDM and to improve care through a quality improvement approach. For example, a standard letter is being piloted for obstetric providers to send to primary care providers after a woman is given a diagnosis of GDM. The consequences of GDM after pregnancy are well-documented, and the findings of our study reinforce the need for public health and primary health care to work together to improve identification and screening of women with prior GDM.
